# Au-Decorated WS_2_/SnO_2_ Heterostructures for Enhanced Room-Temperature NO_2_ Sensing

**DOI:** 10.3390/s26113504

**Published:** 2026-06-02

**Authors:** Myung Sik Choi, Jae-Hun Kim

**Affiliations:** 1 Department of Nano & Advanced Materials Science and Engineering, Kyungpook National University, Sangju 37224, Republic of Korea; ms.choi@knu.ac.kr; 2 Department of Materials Science and Engineering, Inha University, Incheon 22212, Republic of Korea

**Keywords:** SnO_2_ NWs, WS_2_ NSs, Au NPs, NO_2_, RT, gas sensor, sensing mechanism

## Abstract

**Highlights:**

**What are the main findings?**

- Optimized Au-decorated WS_2_/SnO_2_ heterostructures exhibited a high response of 11.7 toward 1000 ppb NO_2_ with an estimated LOD of ~40 ppb at room temperature.
- UV-assisted Au nanoparticle engineering significantly improved the sensing response and selectivity of the optimized 15Au-SW5 sensor.

**What are the implications of the main findings?**

- Synergistic interfacial effects and Au-induced surface modulation contribute to enhanced room-temperature NO_2_ sensing behavior.
- The interface and surface engineering provide a practical strategy for developing low-power, high-performance gas sensors.

**Abstract:**

Nitrogen dioxide (NO_2_) is a highly toxic oxidizing gas; therefore, the development of highly reliable room-temperature (RT) gas sensors with low power consumption is important for practical applications. Herein, WS_2_ nanosheet (NS)–SnO_2_ nanowire (NW) nanocomposites were synthesized and subsequently decorated with Au nanoparticles (NPs) using a UV irradiation method. The SnO_2_ content (1, 5, and 10 wt%) and UV irradiation time (1, 15, and 30 s) were systematically optimized to improve sensing performance. Among the prepared samples, the composite containing 5 wt% SnO_2_ (SW5) exhibited the highest response among the Au-free sensors, while the 15 s UV-treated sample (15Au-SW5) showed a significantly enhanced response of 11.7 toward NO_2_ at RT. The optimized sensor demonstrated reliable ppb-level detection, with an estimated experimental limit of detection of ~40 ppb and good selectivity, repeatability, and long-term stability. The improved performance is considered to be associated with the combined effects of WS_2_–SnO_2_ heterojunctions and Au-induced surface modulation, which may facilitate charge transfer and increase the density of reactive sites. This study highlights that the integration of 2D/1D heterostructures with controlled noble metal decoration is an effective approach for achieving high-performance RT gas sensors.

## 1. Introduction

NO_2_ is a highly toxic oxidizing gas that is widely generated from combustion processes in vehicles, industrial facilities, and power plants [1,2]. Exposure to NO_2_ has been associated with severe respiratory diseases, including asthma and lung damage, and long-term exposure can increase the risk of lung cancer [3,4,5,6,7]. Thus, the threshold limit value (TLV) for NO_2_ is set to 3 ppm. Exposure to NO_2_ even at sub-ppm to ppm levels can adversely affect human respiratory systems and environmental safety, emphasizing the need for highly sensitive NO_2_ detection technologies. In addition, the gas-phase and surface reactions of NO_2_ can be influenced by operating temperature. At elevated temperatures, NO_2_ may partially dissociate into NO and O_2_, which can complicate accurate NO_2_ monitoring in high-temperature sensing systems. Therefore, RT-operated NO_2_ sensors are advantageous for minimizing the influence of thermal dissociation during sensing measurements.

Moreover, it leads to the formation of ozone and acid rain [8]. Furthermore, it is a biomarker for some lung diseases [9]. Thus, realization of sensitive and selective NO_2_ gas sensors with low energy consumption operating at RT is highly important for safety and environmental monitoring.

Resistive gas sensors such as SnO_2_ have high sensitivity, fast dynamics, good stability, and low cost [10,11]. Nonetheless, they often should work at high temperatures, leading to large power consumption, and also have weak selectivity to gases in pristine form [12]. To address these shortages, alternative materials and composite structures have been studied to develop high-performance RT gas sensors [13].

Beyond classical metal oxides, two-dimensional (2D) semiconductors such as transition metal dichalcogenides (TMDs) have a high potential for sensing applications thanks to their large surface area, tunable band gaps, high mechanical flexibility, and high conductivity [14,15]. They have a layered structure with the formula of MX_2_ in which M stands for a transition metal and X represent a chalcogen element [16,17,18]. Among TMDs, WS_2_ has many chemically active sites, a high mobility of charge carriers, and ease of synthesis [19,20]. Nevertheless, pristine WS_2_ often shows weak sensing performance. In this regard, composites between WS_2_ and metal oxides are an effective strategy to improve sensing capacity.

Recently, various heterostructure-based RT NO_2_ gas sensors have been investigated to improve sensing capability through interface engineering and surface modulation. For example, WS_2_/SnO_2_ heterostructures exhibited enhanced RT NO_2_ sensing characteristics, owing to synergistic interfacial charge modulation effects [21]. In addition, vertically aligned MoSe_2_–WS_2_ heterojunctions [22], WS_2_/ZnS heterostructures [23], and Fe/Ni co-doped WS_2_ structures [24] have demonstrated improved NO_2_ sensing performance at RT through enhanced adsorption behavior and interfacial charge transfer. Recent theoretical studies on MoS_2_–WS_2_ heterostructures have also highlighted the importance of interfacial charge redistribution and heterojunction engineering for gas sensing applications [25]. These studies suggest that rational interface engineering is an effective strategy for improving RT NO_2_ sensing performance.

In this context, one-dimensional (1D) SnO_2_ NWs with high stability and excellent charge transfer can be combined with 2D WS_2_ for sensing applications. Creation of WS_2_/SnO_2_ nanocomposites can induce interfacial charge flow and modulation of the electron depletion layer (EDL), leading to enhanced gas response at low operating temperatures [26]. In addition, previous studies have reported that WS_2_/SnO_2_ heterostructures exhibit enhanced room-temperature gas sensing behavior owing to synergistic interfacial interactions and improved charge modulation characteristics [21].

Furthermore, the decoration of noble metal NPs, such as Au, is a good strategy to improve sensing capabilities through the possible formation of Schottky-like junctions at the metal–semiconductor interface, which may modulate charge transport behavior. In addition, Au NPs with high catalytic activity are believed to facilitate oxygen adsorption and activation, thereby increasing the density of reactive oxygen species [27,28,29]. Thus, Au decoration is an effective strategy to boost sensitivity and selectivity of gas sensors [30].

However, despite the extensive development of RT NO_2_ sensors, systematic optimization of WS_2_/SnO_2_ heterostructure composition and UV-assisted Au NP engineering for ppb-level NO_2_ detection remains limited. In particular, the influence of Au NP size and density controlled by UV irradiation time on the sensing behavior of WS_2_/SnO_2_ heterostructures has rarely been investigated. Furthermore, systematic studies on the impact of the heterostructure composition and size of Au NPs on sensing performance are still lacking. Herein, initially WS_2_ NS–SnO_2_ NW formed 2D/1D heterostructures followed by Au NP decoration using a UV reduction route. The SnO_2_ content (1, 5, and 10 wt%) and the UV irradiation time (1, 15, and 30 s) were systematically optimized to enhance sensing performance. The optimized sample exhibited significantly improved NO_2_ sensing characteristics at RT compared to pristine and composite materials. The sensing mechanism is discussed in terms of heterojunction-induced charge modulation and Au-related surface effects, providing insight into the design of high-performance RT gas sensors. For clarity, WS_2_/SnO_2_ composites containing x wt% SnO_2_ are denoted as SWx (x = 1, 5, and 10). Au-decorated samples are denoted as tAu–SW5, where t represents the UV irradiation time (1, 15, and 30 s).

## 2. Materials and Methods

### 2.1. Preparation of SnO_2_ NWs and SnO_2_ NW/WS_2_ NS Composites

**Figure 1:**
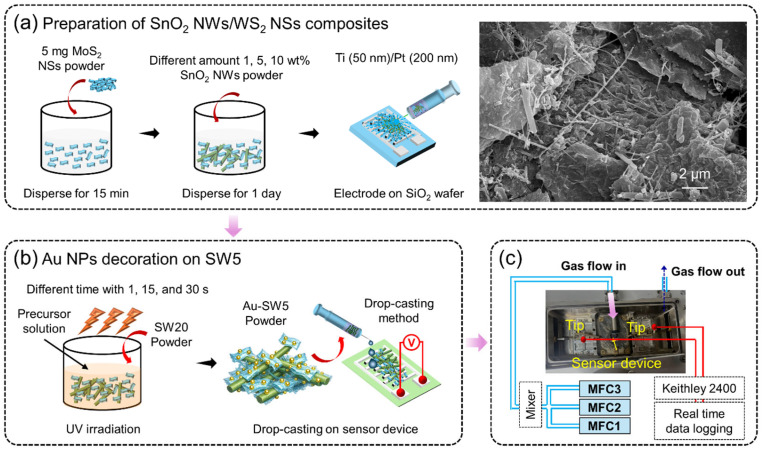
Schematic illustration of (**a**) preparation of WS_2_ NS/SnO_2_ NW composites (SWx), (**b**) Au NP decoration via UV irradiation, and (**c**) the gas sensing measurement system. The inset in (**a**) shows an SEM image of the SW5 sample.

SnO_2_ NWs were synthesized using a vapor–liquid–solid growth process. A thin Au layer (3 nm) was first deposited onto a Si substrate, which was then placed in a quartz tube furnace together with high-purity Sn powder (99.9%, Sigma-Aldrich Co., Ltd., St. Louis, MO, USA). The growth was carried out at 900 °C for 5 min under a mixed gas flow of N_2_ (300 sccm) and O_2_ (10 sccm). After the growth process, the obtained SnO_2_ NWs were collected from the substrate and stored for further use. The synthesized NWs exhibited an average diameter of approximately 50 nm and lengths on the order of tens of micrometers, as schematically illustrated in Figure 1a. For the preparation of WS_2_/SnO_2_ composites, WS_2_ NS powder (5 mg, ACS Material Co., Ltd., Pasadena, CA, USA) was initially dispersed in 2-propanol and sonicated to ensure a homogeneous suspension. Subsequently, different amounts of SnO_2_ NWs (1, 5, and 10 wt%) were added to the solution and mixed thoroughly to form composite suspensions. The prepared mixtures were then drop-cast onto substrates and dried at 80 °C to obtain SWx samples, following the process shown in Figure 1a.

### 2.2. Preparation of Au-Decorated WS_2_–SnO_2_ Nanocomposites

For Au NP decoration, 0.248 mM of HAuCl_6_·H_2_O (KOJIMA chemicals Co., Ltd., Saitama, Japan) was dissolved in 2-propanol. The SnO_2_ NW/WS_2_ NS composites were immersed into the precursor solution and exposed to UV irradiation using a halogen lamp (0.11 mW/cm^2^ and λ = 360 nm) for 1, 15, and 30 s. During UV exposure, Au ions were reduced and decorated onto the surface of the composites (Figure 1b). Subsequently, the samples were annealed at 500 °C for 30 min under N_2_ atmosphere to improve the crystallinity and interfacial contact between the Au NPs and the WS_2_/SnO_2_ heterostructures. As the UV irradiation time increased, the size and surface coverage of Au NPs increased, as confirmed by TEM observations.

### 2.3. Characterizations

The crystalline structure of the samples was analyzed by X-ray diffraction (XRD, Bruker D8 Advance, Billerica, MA, USA) using CuK_α1_ radiation (λ = 1.5406 Å). The morphology and microstructural features were examined by field-emission scanning electron microscopy (FE-SEM, Hitachi S-4200, Tokyo, Japan) and transmission electron microscopy (TEM, JEOL, Tokyo, Japan). The surface chemical composition and elemental states were investigated using X-ray photoelectron spectroscopy (XPS, K-Alpha, Thermo Scientific, Waltham, MA, USA). In addition, ultraviolet photoelectron spectroscopy (UPS, Thermo Fisher Scientific, Theta probe, Waltham, MA, USA) measurements were carried out under ultra-high vacuum conditions (<10^−10^ Torr) with a He I (21.22 eV) excitation source to determine the work function values of the materials.

### 2.4. Gas Sensing Tests

Gas sensing measurements were performed using sensor devices fabricated on substrates with interdigitated electrodes composed of Ti (50 nm) and Au (200 nm) layers. The electrical characteristics of the sensors were monitored using a source meter (Keithley 2400, Cleveland, OH, USA) connected to a computer for real-time data acquisition. The sensors were placed inside a sealed gas chamber with a volume of 517.5 cm^3^, and the overall measurement setup, including gas flow control and electrical monitoring, is illustrated in Figure 1c. The temperature of the system was controlled using a heater located on the backplate. Target gases were introduced into the chamber through mass flow controllers (MFCs), allowing precise control of gas concentration. All gases were balanced with high-purity dry air (99.999%), which was also used for dilution. For example, a certified gas cylinder containing 10 ppm ethanol was diluted with dry air at a ratio of 1:9 to obtain a concentration of 1 ppm. The total gas flow rate was maintained at 500 sccm throughout the measurements. The sensor response was defined based on the resistance change upon gas exposure. For oxidizing gases such as NO_2_, the response was defined as the ratio of resistance in gas (R_g_) to that in air (R_a_), while for reducing gases, the inverse definition (R_a_/R_g_) was used. This response definition is commonly used for resistive gas sensors toward oxidizing gases and enables straightforward comparison with previously reported NO_2_ sensing studies. The dynamic resistance was continuously recorded during alternating exposure to the target gas and air. The response and recovery times were defined as the time required to reach 90% of the final resistance change upon NO_2_ exposure and stoppage, respectively. To evaluate the influence of humidity, humid air was generated by passing dry air through a bubbler system and subsequently mixing it with the target gas using MFCs. The relative humidity (RH) was controlled by adjusting the ratio between dry and humid air, and measurements were conducted under different RH levels ranging from 30% to 90% at room temperature. The humidity values were monitored at 25 °C.

## 3. Results and Discussion

### 3.1. Characterization Studies

**Figure 2:**
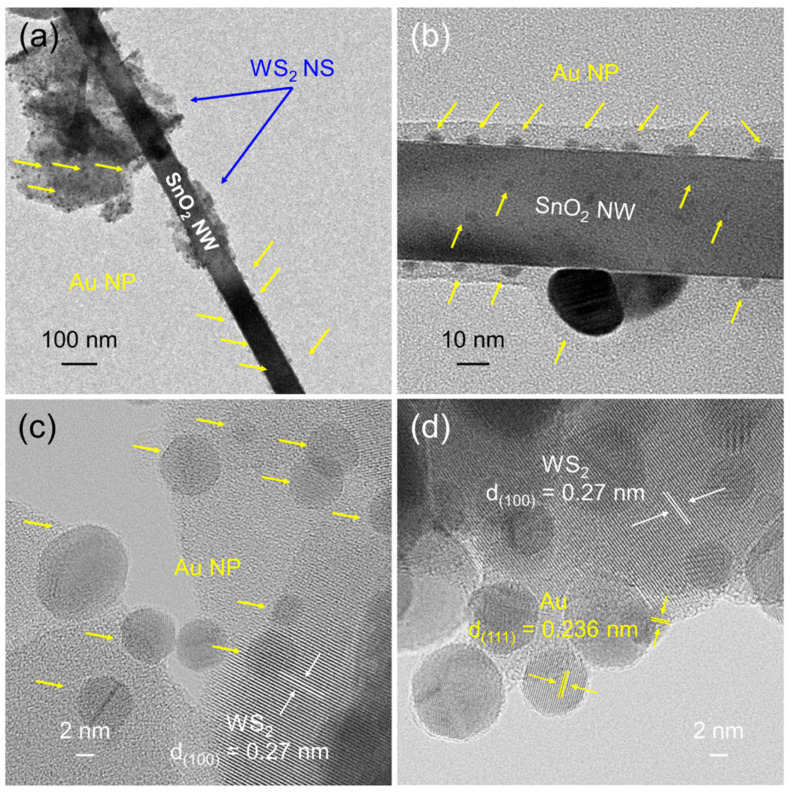
(**a**) TEM image of 15Au-SW5 showing the hybrid structure composed of WS_2_ NSs and SnO_2_ NWs, (**b**) enlarged TEM image showing Au NP distribution, and (**c**,**d**) HRTEM images displaying lattice fringes corresponding to WS_2_ and Au.

Based on the TEM image of SW5 nanocomposite presented in Figure 2a, the nanocomposite formed an interconnected network composed of 1D SnO_2_ NWs intertwined with 2D WS_2_ NSs, which provided efficient charge flow pathways along the NWs while maintaining significant amounts of surface active sites from the NSs. In addition, Figure 2b demonstrates the formation of Au NPs on the surface of the composite, confirming successful decoration of Au NPs. High-resolution TEM images (Figure 2c,d) provide insight into the crystalline nature of the synthesized materials, where lattice fringes with interplanar spacings of 0.27 nm and 0.236 nm are related to the (100) plane of WS_2_ and the (111) plane of Au, respectively. Thus, the coexistence of the WS_2_, SnO_2_, and Au components within the synthesized material was demonstrated.

**Figure 3:**
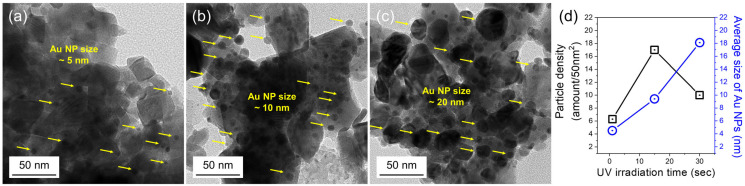
TEM images of Au-decorated SW5 samples prepared with different UV irradiation times: (**a**) 1 s, (**b**) 15 s, and (**c**) 30 s. (**d**) Variations of Au NP density and average Au NP size as a function of UV irradiation time.

The effect of UV irradiation time on Au NP formation was further analyzed using TEM images, as shown in Figure 3a–c. With increasing UV irradiation time from 1 to 30 s, the average Au NP size increased from 4.5 to 18.1 nm. In contrast, the particle density increased from 6.3 to 17 within a 50 nm × 50 nm area when the irradiation time increased from 1 to 15 s, but decreased to 10 after the 30 s irradiation (Figure 3d). This decrease in particle density at prolonged irradiation time is attributed to particle growth and coalescence. Therefore, the 15 s irradiation condition provided the most balanced Au NP size and density, which is favorable for enhancing catalytic activity and interfacial modulation.

**Figure 4:**
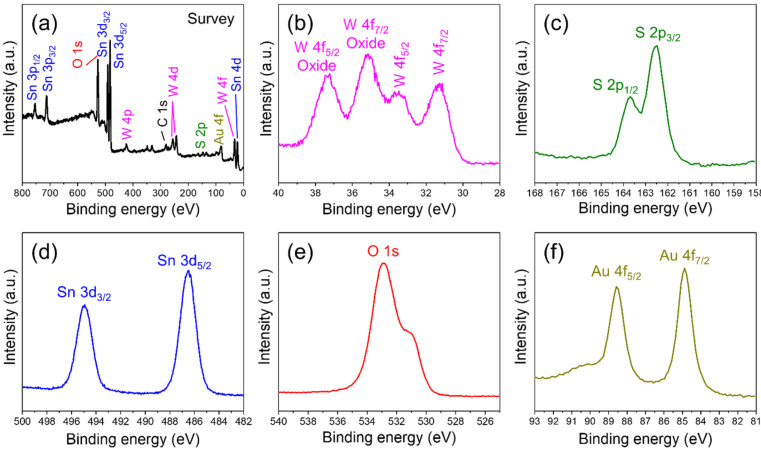
(**a**) XPS survey spectrum of 15Au-SW5, and high-resolution spectra of (**b**) W, (**c**) S, (**d**) Sn, (**e**) O, and (**f**) Au, confirming the chemical states of the WS_2_, SnO_2_, and Au NPs.

The chemical composition and surface states of the optimized sample were analyzed using X-ray photoelectron spectroscopy, as shown in Figure 4. The survey spectrum confirms the presence of W, S, Sn, O, and Au elements, indicating the successful formation of the 15Au-SW5 sample (Figure 4a). The peaks related to Sn, O, W, S, Au, and C (from ambient) were detected, showing high purity of the synthesized sample. Figure 4b offers the W 4f XPS core-level showing the peaks related to W 4f_5/2_ and W 4f_7/2_ belong to WO_3_ which is formed due to negligible oxidation of the WS_2_ surface in air atmosphere along with two peaks related to W 4f_5/2_ and W 4f_7/2_ that belong to the W^4+^ ion in WS_2_ [31]. Figure 4c presents the S 2p core-level region showing two main peak of S 2p_3/2_ and S 2p_1/2_, which belong to the S^2−^ in WS_2_. Figure 4d offers the Sn 3d core-level region with two main peaks of Sn 3d_3/2_ and Sn 3d_5/2_, which belong to the Sn^4+^ in SnO_2_ [32]. Figure 4e gives the O 1s core-level region. It can be related to the presence of the oxygen ion in SnO_2_. Figure 4f presents the Au 4f core-level region with two peaks of Au 4f_5/2_ and Au 4f_7/2_ the belong to metallic Au. Thus, no impurity elements were identified in the synthesized samples.

All in all, the structural and chemical analyses approved the successful synthesis of Au-decorated WS_2_/SnO_2_ heterostructures. The combination of 1D NWs and 2D NSs, along with Au NPs, provides an appropriate platform for enhancing gas sensing output, which is discussed in the following sections.

### 3.2. Gas Sensing Studies

**Figure 5:**
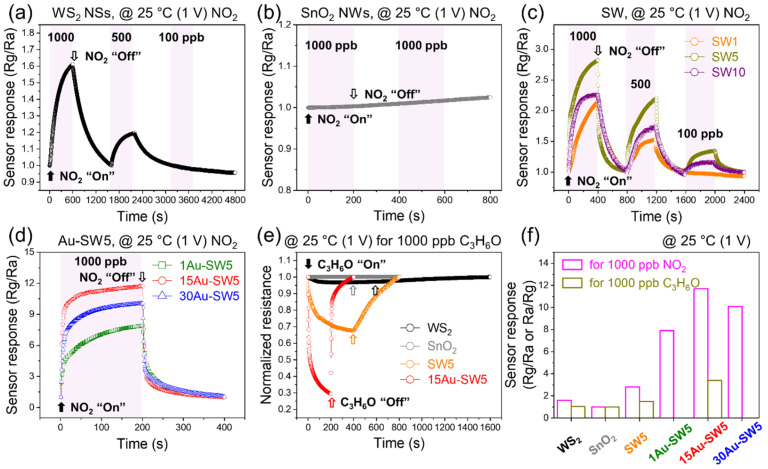
Dynamic sensing responses to NO_2_ at room temperature for (**a**) pristine WS_2_ NSs, (**b**) pristine SnO_2_ NWs, (**c**) SW1, SW5, and SW10 composites, and (**d**) Au-decorated SW5 samples (1Au–SW5, 15Au-SW5, and 30Au–SW5). (**e**) Dynamic responses to acetone and (**f**) comparison of responses to NO_2_ and acetone.

The ppb NO_2_ sensing performance of the fabricated sensors was systematically explored, as shown in Figure 5. As presented in Figure 5a, the pristine WS_2_ sensor exhibited a very weak response to NO_2_, with negligible response at 100 ppb and only a slight increase at higher concentrations. The resistance increased in NO_2_ atmosphere, implying *n*-type sensing behavior of the sensor. In contrast, the pristine SnO_2_ NW sensor (Figure 5b) showed almost no response toward 1000 ppb NO_2_, due to insufficient activation of surface reactions. Next, WS_2_/SnO_2_ nanocomposites with different amounts of SnO_2_ were explored (Figure 5c). All composite sensors (SW1, SW5, and SW10) exhibited improved responses compared to the pristine materials, suggesting the beneficial effect of the heterostructure formation. Among them, the SW5 sensor showed the highest response, with values of 2.1, 2.8, and 2.2 toward 1000 ppb NO_2_ for SW1, SW5, and SW10, respectively. This suggests that an appropriate SnO_2_ content is beneficial for effective interfacial charge modulation at the WS_2_–SnO_2_ interface, while excessive SnO_2_ content may reduce the contribution of WS_2_ active sites. Further improvement was achieved by decorating Au NPs on the SW5 composite (Figure 5d). The sensing response strongly depended on the UV irradiation time used for Au NP formation. Among the tested samples, the 15Au-SW5 sensor exhibited the highest response, reaching approximately 11.7 toward 1000 ppb NO_2_, which is more than four times higher than that of the Au-free SW5 sensor. In contrast, the 1Au–SW5 and 30Au–SW5 sensors showed lower responses, indicating that both insufficient and excessive Au loading are unfavorable. This behavior can be attributed to the balance between catalytic activity and effective Schottky junction formation, which is optimized at an intermediate Au NP size and density. The selectivity of the sensors was evaluated by comparing their responses to NO_2_ and acetone (Figure 5e,f). While all sensors showed some response to acetone, the 15Au-SW5 sensor exhibited a significantly higher response to NO_2_, demonstrating preferential sensitivity toward oxidizing gases under the present conditions. The response and recovery times of the optimized 15Au-SW5 sensor toward 1000 ppb NO_2_ gas were estimated to be 37 and 28 s, respectively. The relatively fast sensing dynamics are associated with the enhanced surface reactions and interfacial charge modulation induced by the WS_2_/SnO_2_ heterostructure and Au NP decoration.

**Figure 6:**
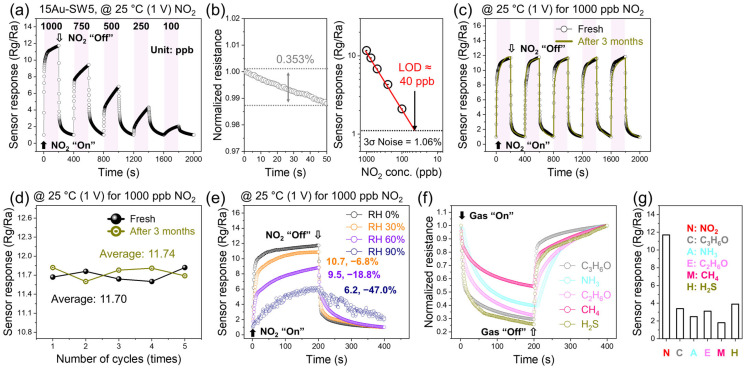
Gas sensing characteristics of the optimized 15Au-SW5 sensor (**a**) Dynamic sensing transients toward 100–1000 ppb NO_2_ gas at RT. (**b**) Baseline noise analysis and estimated LOD. (**c**) Dynamic sensing curves toward 1000 ppb NO_2_ in fresh state and after 3 months. (**d**) Long-term stability toward 1000 ppb NO_2_ after 3 months. (**e**) Sensing characteristics toward 1000 ppb NO_2_ under different RH conditions. (**f**) Normalized sensing transients toward 1000 ppb of different gases. (**g**) Corresponding selectivity histogram.

The sensing features of the optimized 15Au-SW5 sensor are indicated in Figure 6. As shown in Figure 6a, the sensor manifested reversible resistance changes when exposed to different ppb levels of NO_2_ at RT. The response values were 2.1, 4.3, 6.8, 9.4, and 11.7 for 100, 250, 500, 750, and 1000 ppb NO_2_, respectively, showing a monotonic increase with concentration. The corresponding calibration curve is linear in the measured range (Figure 6b). Experimentally, the sensor revealed a limit of detection (LOD) of 40 ppb with a response of 1.1, while theoretical LOD was calculated to be ~1 ppb. The baseline noise, signal stability, and the limited resolution of low-concentration measurements attributes to difference between the experimental and theoretical LOD values. The repeatability and long-term stability of the 15Au-SW5 sensor were also investigated. As shown in Figure 6c,d, the sensor exhibited consistent responses over sequential cycles, with average response values of 11.70 and 11.74 for fresh and preserved sensors, respectively, indicating excellent stability along with repeatability.

The effect of humidity was explored by monitoring the sensing response under different RH conditions (Figure 6e). The response values decreased 6.8%, 18.8%, and 47% relative to dry condition, at RH levels of 30, 60, and 90%. Thus, the sensing response gradually decreased with increasing RH, indicating competitive adsorption between H_2_O molecules and NO_2_ molecules on active sensing sites. Nevertheless, distinguishable sensing behavior was maintained even under high humidity conditions. Under humid conditions, adsorbed H_2_O molecules may occupy active adsorption sites and interfere with the adsorption of NO_2_ molecules and reactive oxygen species on the sensor surface. As a result, interfacial charge transfer associated with NO_2_ sensing can be partially suppressed, leading to reduced sensing response at high RH conditions. Finally, the selectivity of the optimized sensor was investigated towards various gases namely, NO_2_, acetone, ethanol, ammonia, methane, and hydrogen sulfide (Figure 6f). The response values were 11.7, 3.4, 2.5, 3.1, 1.8, and 3.9, respectively, demonstrating good selectivity of the gas sensor towards NO_2_ gas **(**Figure 6g).

A comparison of the sensing performance with previously reported RT NO_2_ gas sensors is summarized in Table 1, demonstrating the competitive sensing characteristics of the present 15Au-SW5 sensor.

### 3.3. Gas Sensing Mechanism

The sensing mechanism of gas sensors in this work can be explained using surface adsorption reactions, heterojunction effects, and an Au catalytic effect.

Initially, in air, oxygen molecules capture the electrons from the sensor’s surface, forming ionized oxygen species. This leads to the formation of an electron depletion layer (EDL) with low amount of electrons relative to the core region. At RT, adsorbed oxygen species are mainly present in molecular or weakly ionized forms. In the presence of NO_2_ gas, additional electrons are extracted from the sensor surface, leading to expansion of the electron depletion layer and an increase in sensor resistance. However, in pristine sensors, the number of resistance modulation sources were limited, eventually leading to a low response.

**Figure 7:**
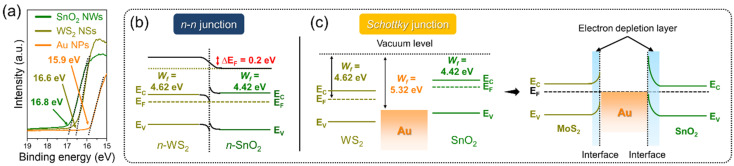
(**a**) UPS spectra and (**b**) energy band alignment of WS_2_, SnO_2_, and Au, and (**c**) schematic illustration of *n*–*n* heterojunction formation between WS_2_ NSs and SnO_2_ NWs.

In WS_2_/SnO_2_ nanocomposite sensors, the formation of *n*–*n* heterojunctions should be considered. Based on the UPS results (Figure 7a), the work function values of WS_2_ and SnO_2_ were estimated to be 4.62 eV and 4.42 eV, respectively. Thus, in intimate contact between the two materials, electrons transfer from SnO_2_ to WS_2_ to equate the Fermi levels, resulting in band bending at the interface and potential barriers for the flow of electrons (Figure 7b). Upon exposure to NO_2_ and further extraction of electrons, the height of potential barriers increased, leading to remarkable resistance, contributing to a large response. This explains the improved sensing performance observed for nanocomposite sensors.

**Figure 8:**
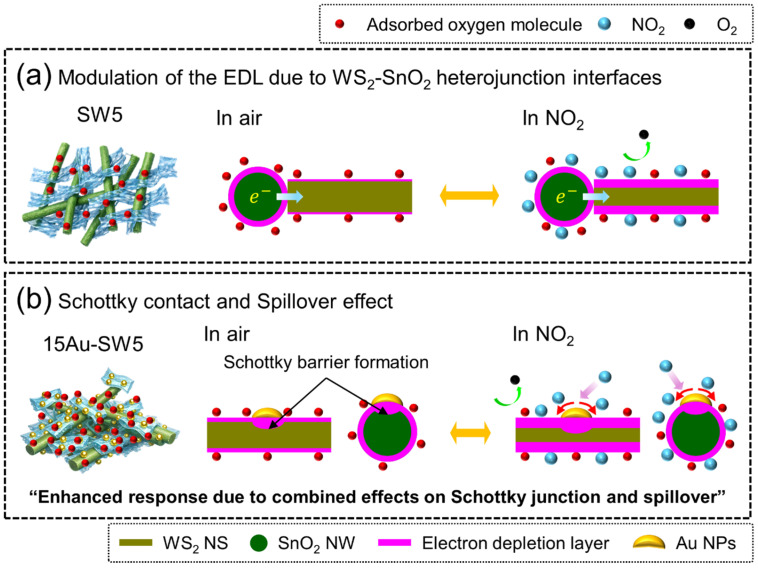
Schematic illustration of the NO_2_ sensing mechanism: (**a**) WS_2_/SnO_2_ heterostructure showing modulation of the electron depletion layer and (**b**) Au-decorated system highlighting Schottky barrier formation and spillover-assisted surface reactions.

For Au-decorated samples, additional effects contribute to the sensing enhancement (Figure 8). First, Au NPs act as catalytic sites that facilitate the adsorption and activation of oxygen molecules. The activated oxygen species can migrate onto the surface of WS_2_ and SnO_2_ through a spillover process, increasing the density of reactive sites. Second, due to the higher work function of Au (5.32 eV), electrons transfer from the semiconductor materials to the Au NPs, resulting in the formation of Schottky barriers at the Au–WS_2_ and Au–SnO_2_ interfaces. These barriers introduce additional depletion regions and increase the sensitivity of the sensor to changes in surface charge.

When NO_2_ gas is introduced, the combined effects of heterojunction modulation and Au-induced Schottky barriers lead to a more pronounced expansion of the depletion region and a larger change in resistance. The sensing performance therefore depends on the amount and size of Au NPs. At low Au loading, the number of catalytic sites and Schottky junctions is insufficient. In contrast, excessive Au loading leads to particle aggregation, which reduces the effective surface area and the number of active junctions. As a result, an optimal condition is achieved for the 15Au-SW5 sensor, where both catalytic activity and interfacial charge modulation are maximized.

Overall, the enhanced sensing performance of the optimized sensor can be attributed to the synergistic effects of WS_2_–SnO_2_ heterojunctions and Au-induced surface modulation, which together enable efficient charge transfer and amplified resistance variation upon NO_2_ exposure.

## 4. Conclusions

WS_2_ NSs were hybridized with SnO_2_ NWs and further decorated with Au NPs via controlled UV irradiation to achieve enhanced NO_2_ sensing at RT. The systematic tuning the amount of SnO_2_ and Au NP characteristics strongly affected the sensing performance. Among the fabricated sensors, the optimized 15Au-SW5 sensor exhibited a high response of 11.7 toward 1000 ppb NO_2_ with an estimated experimental LOD of ~40 ppb, along with good selectivity and long-term stability, demonstrating its potential for practical NO_2_ sensing applications. The enhanced sensing behavior was related to the formation of WS_2_–SnO_2_ heterojunctions, Au-induced interfacial modulation, and the catalytic surface reactions of the Au NPs. These results suggest that simultaneous optimization of heterostructure composition and Au NP characteristics is an effective strategy for achieving highly sensitive ppb-level NO_2_ sensing at RT.

## Figures and Tables

**Table 1:** Comparison of RT NO_2_ sensing performance of recently reported heterostructure-based gas sensors.

Sensing Material	Conc. (ppm)	Response (R_g_/R_a_ or R_a_/R_g_)	LOD (ppb)	τ_Res_/τ_Rec_ (s)	Stability	Ref.
15Au-SW5	1	11.7	40	37/28	90 days	This work
2D/0D WS_2_/SnO_2_ (UV light)	10	4	50	9/8	30 days	[21]
MoSe_2_-WS_2_ nanoworms	0.05	59.6% ^1^	50	69/66	60 days	[22]
MXene–Cu_2_O composite	0.5	6.07	10	55/35	30 days	[33]
Zn-doped Cu_2_O/CuO	10	30.3	2	35/47	30 days	[34]
SnS_2_/Si heterostructure	40	671% ^1^	171	33/402	30 days	[35]
MoSe_2_ nanoroses and rGO composite	4	38% ^1^	100	110/128	60 days	[36]

^1^ Note: R(%) = [ΔR/R_a_] × 100.

## Data Availability

Data will be made available on request.
